# Poly(beta-amino ester) nanoparticles enable tumor-specific TRAIL secretion and a bystander effect to treat liver cancer

**DOI:** 10.1016/j.omto.2021.04.004

**Published:** 2021-04-16

**Authors:** Hannah J. Vaughan, Camila G. Zamboni, Nicholas P. Radant, Pranshu Bhardwaj, Esther Revai Lechtich, Laboni F. Hassan, Khalid Shah, Jordan J. Green

**Affiliations:** 1 Department of Biomedical Engineering, Institute for NanoBioTechnology, and the Translational Tissue Engineering Center, Johns Hopkins University School of Medicine, Baltimore, MD 21231, USA; 2 Center for Stem Cell Therapeutics and Imaging, Department of Neurosurgery, Brigham and Women’s Hospital, Harvard Medical School, Boston, MA 02115, USA; 3 Departments of Ophthalmology, Oncology, Neurosurgery, Materials Science & Engineering, and Chemical & Biomolecular Engineering, and the Bloomberg∼Kimmel Institute for Cancer Immunotherapy, Johns Hopkins University School of Medicine, Baltimore, MD 21231, USA

**Keywords:** gene therapy, gene delivery, TRAIL, liver cancer, nanoparticle, bystander effect, histone deacetylase inhibitors, polymer, PBAE

## Abstract

Despite initial promise, tumor necrosis factor-related apoptosis-inducing ligand (TRAIL)-based approaches to cancer treatment have yet to yield a clinically approved therapy, due to delivery challenges, a lack of potency, and drug resistance. To address these challenges, we have developed poly(beta-amino ester) (PBAE) nanoparticles (NPs), as well as an engineered cDNA sequence encoding a secretable TRAIL (sTRAIL) protein, to enable reprogramming of liver cancer cells to locally secrete TRAIL protein. We show that sTRAIL initiates apoptosis in transfected cells and has a bystander effect to non-transfected cells. To address TRAIL resistance, NP treatment is combined with histone deacetylase inhibitors, resulting in >80% TRAIL-mediated cell death in target cancer cells and significantly slowed xenograft tumor growth. This anti-cancer effect is specific to liver cancer cells, with up to 40-fold higher cell death in HepG2 cancer cells over human hepatocytes. By combining cancer-specific TRAIL NPs with small-molecule-sensitizing drugs, this strategy addresses multiple challenges associated with TRAIL therapy and offers a new potential approach for cancer treatment.

## Graphical abstract

**Figure:**
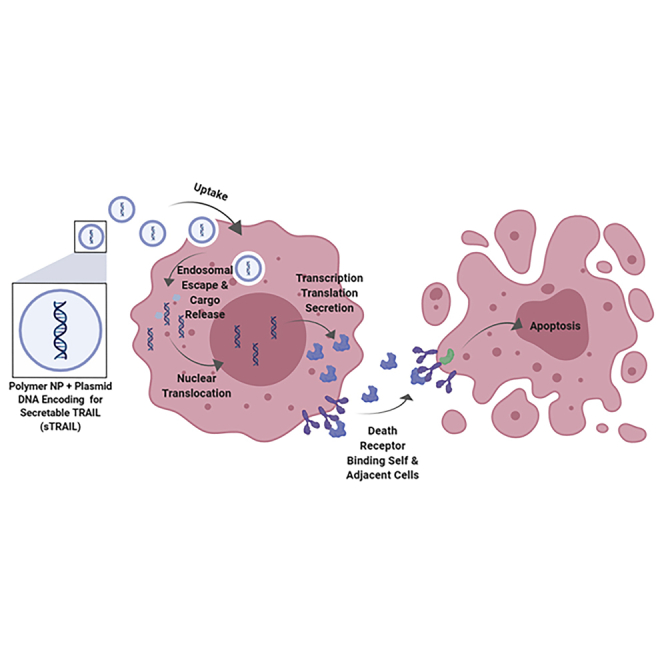

## Teaser

Green and colleagues develop polymeric nanoparticles to program liver cancer cells to locally secrete tumor necrosis factor-related apoptosis-inducing ligand (sTRAIL). The authors show that the delivered sTRAIL initiates apoptosis in transfected liver cancer cells and has a bystander effect to non-transfected cancer cells, with potency enhanced by histone deacetylase inhibitors.

## Introduction

Tumor necrosis factor-related apoptosis-inducing ligand (TRAIL) is a protein ligand that has been studied for over two decades as an anti-cancer agent.^1^^,^^2^ Upon TRAIL binding to death receptors DR4 and DR5, intracellular death domains cluster and initiate apoptotic signaling via assembly of the death-inducing signaling complex (DISC).^3^ DR4 and DR5 are overexpressed in many cancers, while healthy cells overexpress decoy receptors DcR1 and DcR2, which bind TRAIL protein but do not contain fully functional intracellular death domains required for apoptosis.^4^^,^^5^ These differences in death receptor expression, as well as abnormal regulation of apoptotic signaling, result in TRAIL initiating apoptosis selectively in cancer cells with limited toxicity to healthy cells and tissues.^6^

Although TRAIL has shown promising therapeutic effects *in vitro* and in animal cancer models, it has failed to show significant anti-tumor efficacy in clinical trials.^7^^,^^8^ Recombinant TRAIL protein is rapidly cleared, with a serum half-life of approximately 30 min.^9^ This results in low TRAIL accumulation in the tumor, likely underpinning the lack of robust anti-tumor response. Additionally, there is evidence of acquired and innate TRAIL resistance in many tumor types, which has inspired investigation into combination therapies and sensitizing agents.^10^

Lackluster clinical efficacy has motivated gene therapy approaches to improve TRAIL-based cancer treatment.^11^ TRAIL gene therapy directly delivers TRAIL-encoding cDNA to cancer cells, enabling cytokine production locally in the tumor. This approach maximizes the local concentration of TRAIL protein, while minimizing systemic exposure and toxicity. Several groups have employed viral vectors for TRAIL gene therapy and achieved efficient suppression of xenograft tumor growth in various cancer types.^12^, ^13^, ^14^ However, there are safety concerns inherent to viral gene therapy, including risk of immunogenicity,^15^ tumorigenicity,^16^ and cytotoxicity,^17^ as well as the practical limitations of limited cargo carrying capacity and manufacturing challenges.

Non-viral gene delivery systems are generally safe and non-immunogenic but often have lower delivery efficacy than their viral counterparts.^18^ To address this limitation, we have developed cDNA encoding a secretable form of TRAIL, which we deliver to hepatocellular carcinoma (HCC) using poly(beta-amino ester) PBAE nanoparticles (NPs). We explored combining this TRAIL NP therapy with histone deacetylase (HDAC) inhibitors, which have shown promise in sensitizing resistant cells to TRAIL.^19^, ^20^, ^21^, ^22^, ^23^ We hypothesized that the bystander effect of secreted TRAIL to non-transfected cells combined with HDAC inhibitor sensitization could result in a potent yet cancer-specific non-viral TRAIL gene therapy.

## Results

### PBAE NPs enable DNA delivery to HepG2 *in vitro* and *in vivo*

**Figure 1:**
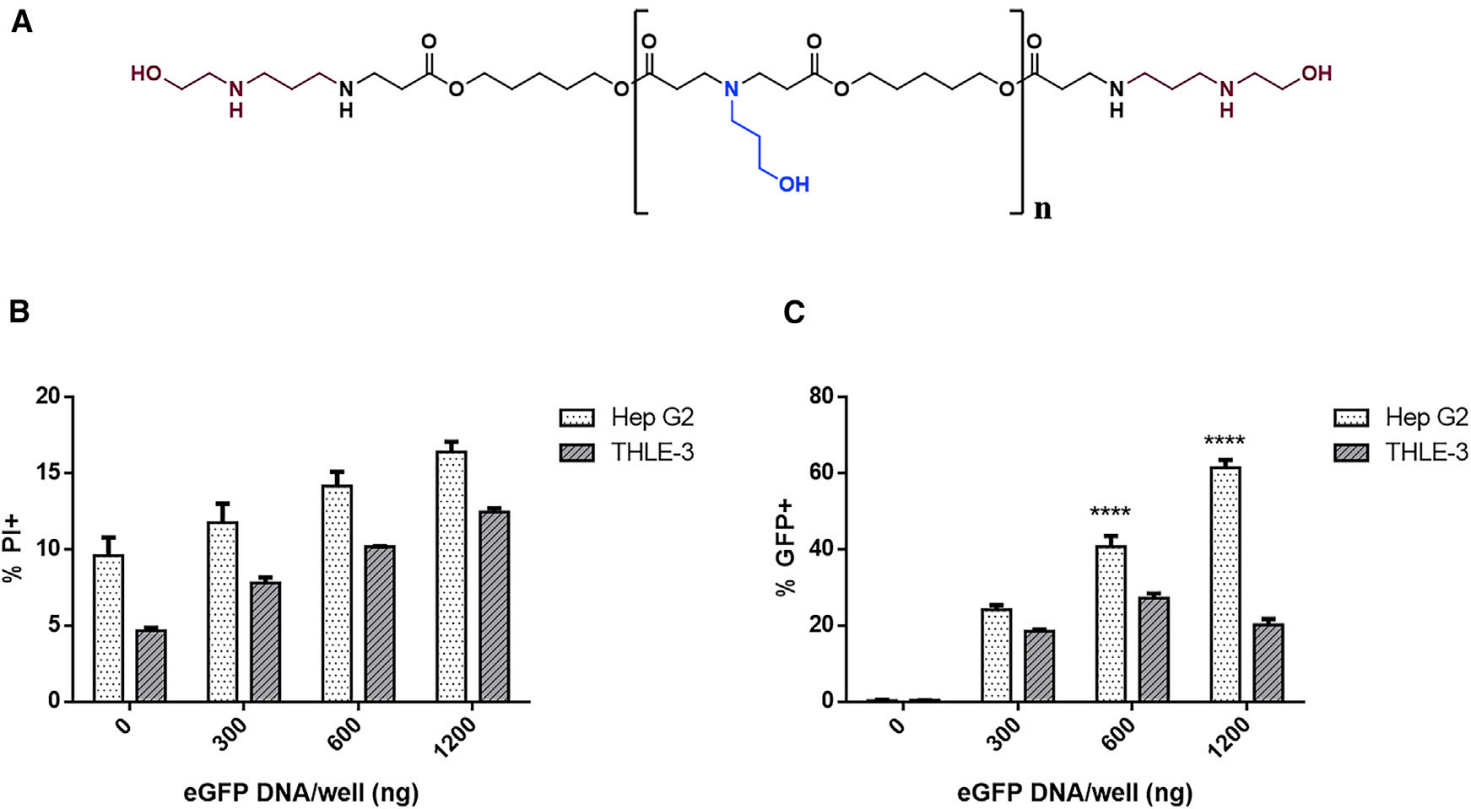
PBAE 536 NPs enable selective intracellular delivery of a reporter gene to HepG2 Cells *in vitro* (A) Chemical structure of polymer 2-((3-aminopropyl)amino)ethanol end-modified poly(1,5-pentanediol diacrylate-co-3-amino-1-propanol) (PBAE 536). (B) Viability of HepG2 and THLE3 cells after treatment with PBAE 536 NPs at a range of eGFP DNA doses. Toxicity was determined by staining samples 1:200 with propidium iodide (PI) and measuring the percentage of PI^+^ cells by flow cytometry. (C) *In vitro* eGFP transfection of HepG2 HCC cells and THLE3 hepatocytes by PBAE 536 NPs measuring the percentage of GFP^+^ cells by flow cytometry. Data represent mean ± SEM of three replicate wells. Statistically significant differences in transfection between HepG2 and THLE3 determined using two-way ANOVA and Sidak’s multiple comparisons test. *∗∗∗∗*p < 0.0001.

PBAEs are a class of biodegradable polyesters that have been employed for nucleic acid delivery to a wide range of cell types.^24^ To form DNA NPs, PBAE cationic polymer is combined with anionic plasmid DNA at varying weight/weight (w/w) ratios, and the polyelectrolytes self-assemble electrostatically into polyplexes. These NPs facilitate efficient cellular uptake, endosomal escape, and expression of the encapsulated gene cargo. Our lab has shown that by varying the composition of PBAE polyplexes, we can tune transfection efficacy in a wide range of cell types, while minimizing NP cytotoxicity.^25^^,^^26^ Notably, we recently used high-throughput screening to optimize DNA delivery to an array of nine HCC cells lines and identified a polymer termed PBAE 536 (Figure 1A) as the superior candidate for gene delivery across these cell lines.^27^ Therefore, we selected PBAE 536 NPs as a non-viral DNA delivery vehicle for HCC cells in this study.

PBAE NPs were characterized and evaluated for DNA delivery in both HCC cells and healthy human hepatocytes. PBAE 536 was synthesized via Michael addition in a two-step reaction (Figure S1). To form NPs, we combined PBAE 536 with eGFP-N1 plasmid DNA at 25 w/w and allowed to self-assemble in sodium acetate (pH = 5). Electrostatic interactions between the cationic polymer and anionic nucleic acid facilitated the formation of NPs with a hydrodynamic diameter of ∼200 nm and a zeta potential of +16 mV. Gel electrophoresis was performed to quantify DNA encapsulation efficiency as a function of w/w ratio. Encapsulation efficiency was ∼100% for all formulations tested (Figure S2). Dynamic light scattering (DLS) measurements were performed on NPs formulated with various plasmid DNAs, as well as PBAE polymer alone. We determined that NP size is independent of the plasmid sequence, and there is a significant decrease in particle size in the absence of plasmid DNA, demonstrating that electrostatic complexation drives the formation of the NPs (Figure S3). To evaluate these NPs for gene delivery, *in vitro* cultures of HepG2 human HCC cells and THLE-3 healthy human hepatocytes were incubated with varying doses of eGFP-N1 PBAE 536 NPs. Increasing doses of NPs caused increased toxicity in both HepG2 and THLE-3 cells (Figure 1C). Transfection efficacy was also dose-dependent, with increasing transfection with higher DNA doses in both cell types (Figure 1D). An intermediate dose of 600 ng DNA per well was selected for further studies because this was the lowest dose tested with significantly increased transfection in HepG2 HCC cells over THLE-3 hepatocytes. At this dose, toxicity was maintained below 15% for both cell types, and transfection rates were 41% ± 3% and 27% ± 1% in HepG2 and THLE-3 cells, respectively. Therefore, PBAE 536 NPs enable specific transfection of HepG2 cells over healthy hepatocytes, without toxicity to either cell line. HepG2 cells transfected with empty pN3 backbone plasmid showed similar toxicity profile to eGFP transfected cells and no significant fluorescence, indicating that transgene expression does not cause significant toxicity or background fluorescence in transfected cells (Figure S4). These results confirm a biomaterial-mediated cancer specificity of PBAE 536 NPs that has been previously reported.^28^

### PBAE NP transfection with sTRAIL plasmid results in TRAIL protein secretion

**Figure 2:**
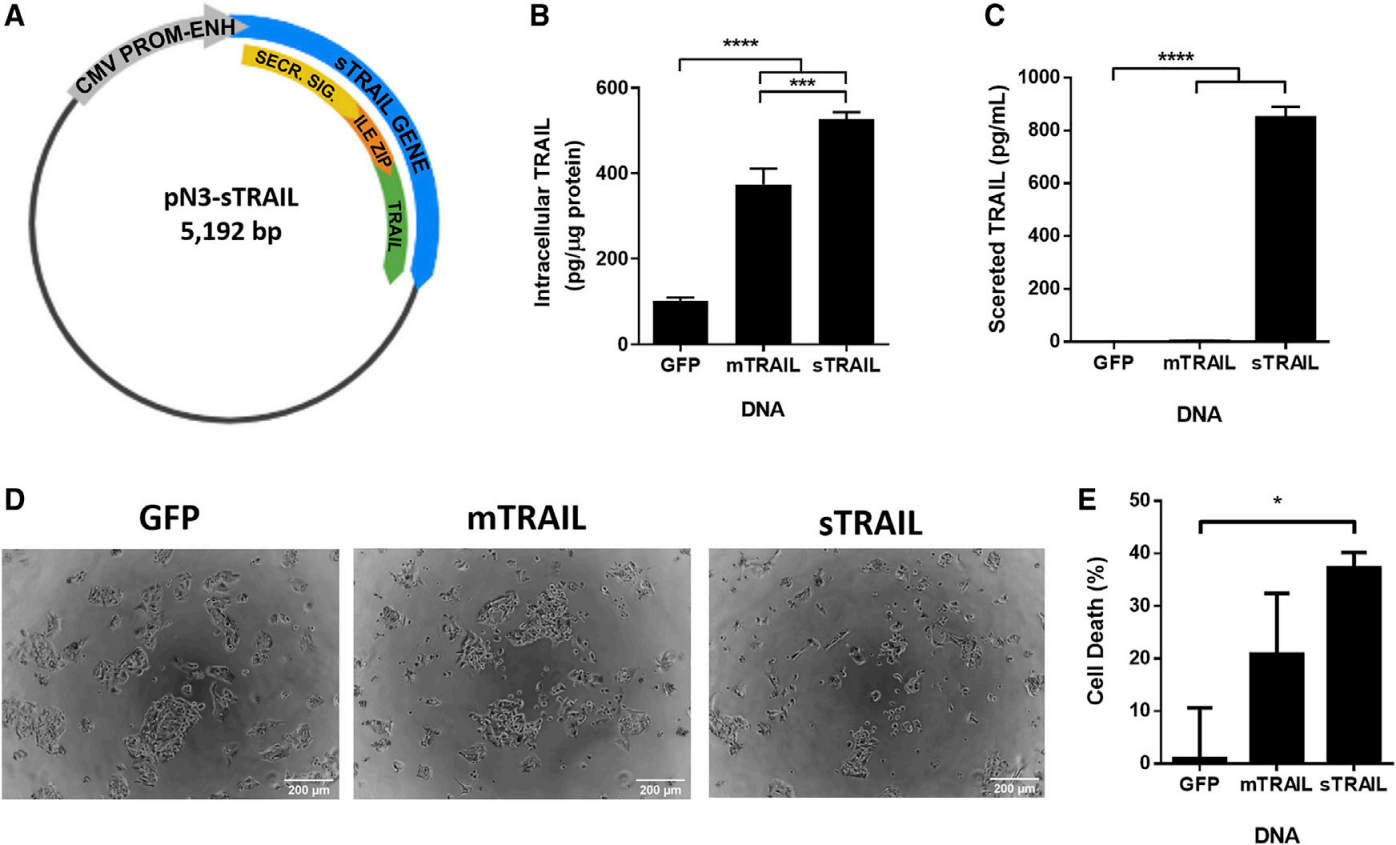
Transfection with PBAE 536 NPs carrying the sTRAIL plasmid results in production and secretion of human TRAIL protein (A) Map of the engineered sTRAIL plasmid. (B and C) Intracellular (B) and secreted (C) human TRAIL protein in HepG2 cells measured by ELISA after PBAE 536 NP treatment. (D) Representative phase contrast images of HepG2 cells after transfection with PBAE 536 NPs containing eGFP, mTRAIL, or sTRAIL plasmid DNA. Scale bar, 200 μm. (E) Treatment-mediated cell death in HepG2 cells measured by MTT, expressed as a percentage of metabolic activity normalized to untreated HepG2 cells. All data represented as mean ± SEM of n = 3 replicate wells. Significant differences between groups determined by one-way ANOVA and Tukey post hoc test. *∗∗∗∗*p < 0.0001, ∗∗∗p < 0.001, ∗p < 0.05.

With the aim of developing a TRAIL gene therapy with a potent bystander effect, we engineered a secretable TRAIL (sTRAIL) plasmid (Figure 2A). The non-viral sTRAIL construct, based on a viral construct developed by Shah et al.,^29^ comprises three components: (1) a secretion signal derived from the extracellular domain of Flt3L, a ligand for the Flt tyrosine kinase receptor involved in protein secretion, (2) an isoleucine zipper trimerization domain to facilitate the assembly of a biologically active TRAIL homotrimer, and (3) the apoptosis-inducing sequence derived from the N terminus of the human TRAIL sequence. The coding sequences of these three domains were combined and inserted into the multiple cloning site of the pN3 backbone downstream of the cytomegalovirus promoter-enhancer sequence.^30^ The full cDNA sequence can be found in Figure S5 (Addgene #154246). As a positive control, we utilized a plasmid encoding for the endogenous transmembrane human TRAIL protein in a pEGFP-C3 backbone, which we refer to as mTRAIL (membrane TRAIL) to differentiate it from sTRAIL.^31^^,^^32^

Next, we evaluated TRAIL protein expression and secretion in cells transfected with sTRAIL and mTRAIL plasmids. PBAE 536 NPs were fabricated with mTRAIL or sTRAIL DNA, and these NPs were used to transfect HepG2 cells. After 48 h, lysates and media samples were collected from transfected cells, and an ELISA for human TRAIL was performed on these samples. These results showed that cells treated with sTRAIL-NPs had intracellular TRAIL expression of 530 pg per μg of total protein, an expression level similar to cells treated with the mTRAIL positive control plasmid (Figure 2B). However, there were striking differences in the conditioned cell culture media from mTRAIL and sTRAIL transfected cells. sTRAIL-treated cells secreted TRAIL protein extracellularly, with a concentration of 850 pg/mL after 48 h (Figure 2C). However, cells transfected with mTRAIL showed no detectable protein secretion. Taken together, these results confirm that the sTRAIL sequence developed for these studies encodes for human TRAIL protein, as detected by ELISA. Additionally, the modifications made in the engineered sTRAIL cDNA enable secretion of this TRAIL protein.

We next measured cellular viability to confirm that the protein secreted by sTRAIL transfected cells maintained the pro-apoptotic function of TRAIL. After 24 h, HepG2 cells treated with sTRAIL NPs are sparser, rounded, and form smaller clumps than cells transfected with control eGFP-N1 NPs (Figure 2D). By 3-(4,5-dimethylthiazol-2-yl)-2,5-diphenyltetrazolium bromide (MTT) assay, the viability of sTRAIL transfected cells was reduced by 37% over untreated cells, while the loss in viability from control GFP NPs was only 1% (Figure 2E). Transfection with mTRAIL showed only 21% decrease in viability, although there is not a statistically significant difference in comparison to sTRAIL transfected cells. These results suggest that the modifications to the sTRAIL sequence did not mitigate the anticancer effect of TRAIL protein.

### HDAC inhibitors sensitize HepG2 cells to TRAIL-induced apoptosis

**Figure 3:**
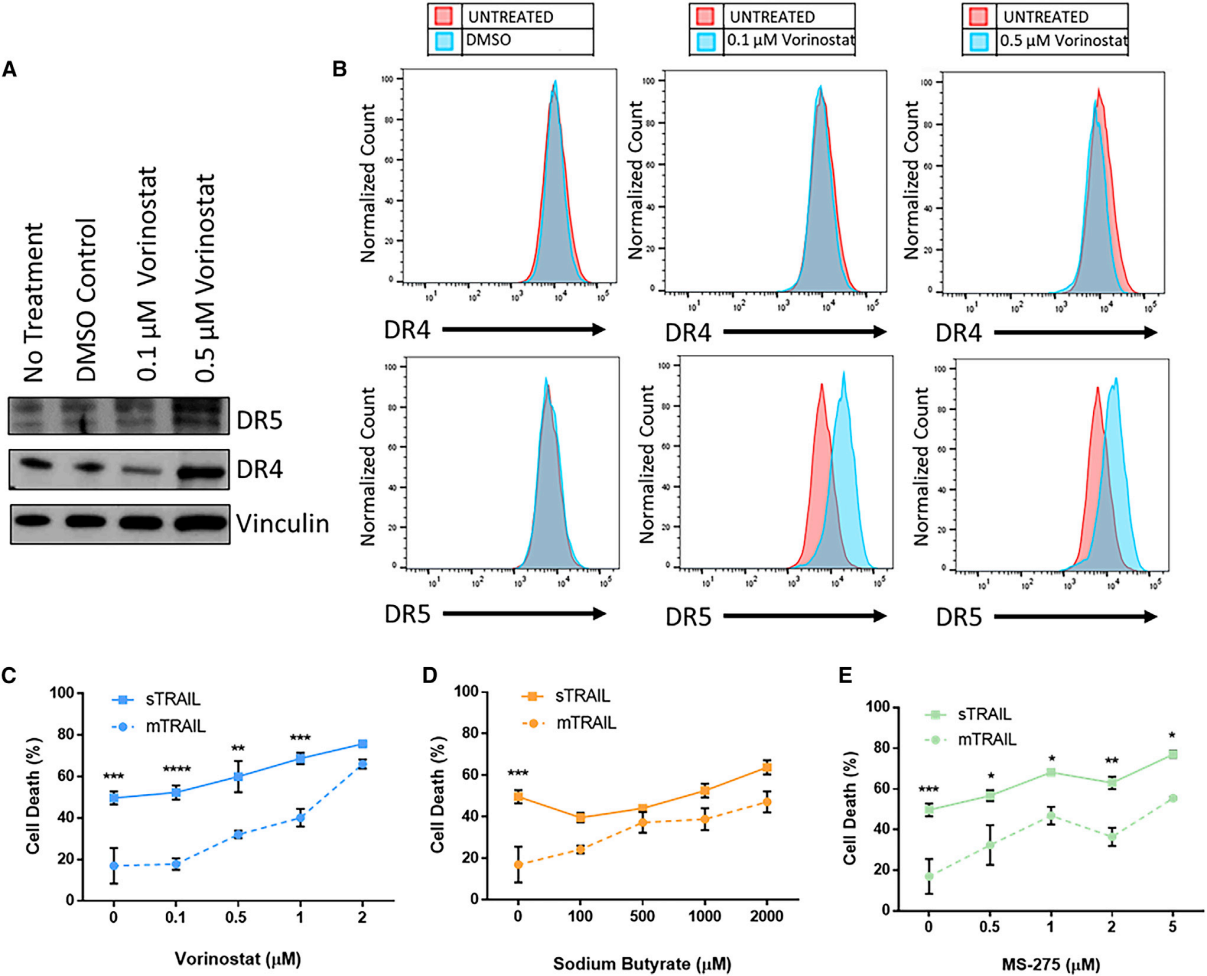
HDAC Inhibitors cause upregulation of death receptor expression and sensitize HepG2 cells to TRAIL NPs (A and B) HepG2 death receptor (DR4 and DR5) expression by (A) western blot and (B) flow cytometry after 24-h vorinostat treatment. (C–E) HepG2 cell viability 48 h after transfection with either mTRAIL or sTRAIL NPs and treated with varying doses of (C) vorinostat, (D) sodium butyrate, and (E) MS-275. Data represented as mean ± SEM of n = 3 replicate wells. Significant differences between sTRAIL and mTRAIL-treated cells are determined by two-way ANOVA and Sidak’s multiple comparison test. *∗*p < 0.05, ∗∗p < 0.01, ∗∗∗p < 0.001, ∗∗∗p < 0.0001.

HDAC inhibitors have been explored as a cancer treatment as a monotherapy and in combination with chemotherapy or radiation.^33^ This class of drugs acts by inhibiting histone deacetylases, effectively opening chromatin and affecting gene expression at the epigenetic level, including key tumor suppressors and resistance genes.^34^ Some studies have also shown synergistic anti-cancer effects between TRAIL and HDAC inhibitors.^35^, ^36^, ^37^ Western blot analysis shows that the HDAC inhibitor vorinostat alters death receptor expression in HepG2 cells (Figure 3A). After 24-h treatment with vorinostat, DR4 and DR5 expression are increased. Flow cytometry confirms that surface expression of DR5 increases with vorinostat exposure in a dose-dependent manner (Figure 3B). However, surface DR4 is unchanged by vorinostat exposure, suggesting that trafficking of death receptors to the cell membrane remains a barrier. Receptor mutation studies have shown that DR5 has a greater contribution to TRAIL mediated apoptosis than DR4, so we hypothesized that increased DR5 expression alone could mediate a significant sensitizing effect.^38^, ^39^, ^40^

We combined PBAE 536 sTRAIL NP treatment with low doses of three HDAC inhibitors as sensitizing agents: vorinostat, sodium butyrate, and MS-275. HepG2 cells were incubated with NPs for 2 h, then sensitizers were added. While HDAC inhibitors have shown promise as anti-cancer agents, we used low doses with limited toxicity to cancer cells when used alone (Figure S6). After 48 h, viability was measured by MTT assay, and treatment wells were normalized to wells treated with control GFP NPs and the same sensitizer dose to isolate TRAIL-mediated apoptosis.

HepG2 cells treated with HDAC inhibitors showed higher TRAIL-mediated cell death, compared with TRAIL NP treatment alone (Figures 3C–3E). This sensitizing effect was dose-dependent, with higher HDAC inhibitor concentration resulting in >70% loss in viability. This suggests that the increased surface DR5 expression from HDAC inhibitor treatment effectively sensitize HepG2 cells to TRAIL-mediated cell death. Further, combination treatment with sTRAIL NPs was more potent than with mTRAIL NPs at most of the HDAC inhibitor doses tested. This demonstrates that the bystander effect enabled by TRAIL secretion increases the potency of TRAIL gene therapy.

### Combination treatment of sTRAIL PBAE NPs and HDAC inhibitors shows cancer-specific apoptosis *in vitro*

**Figure 4:**
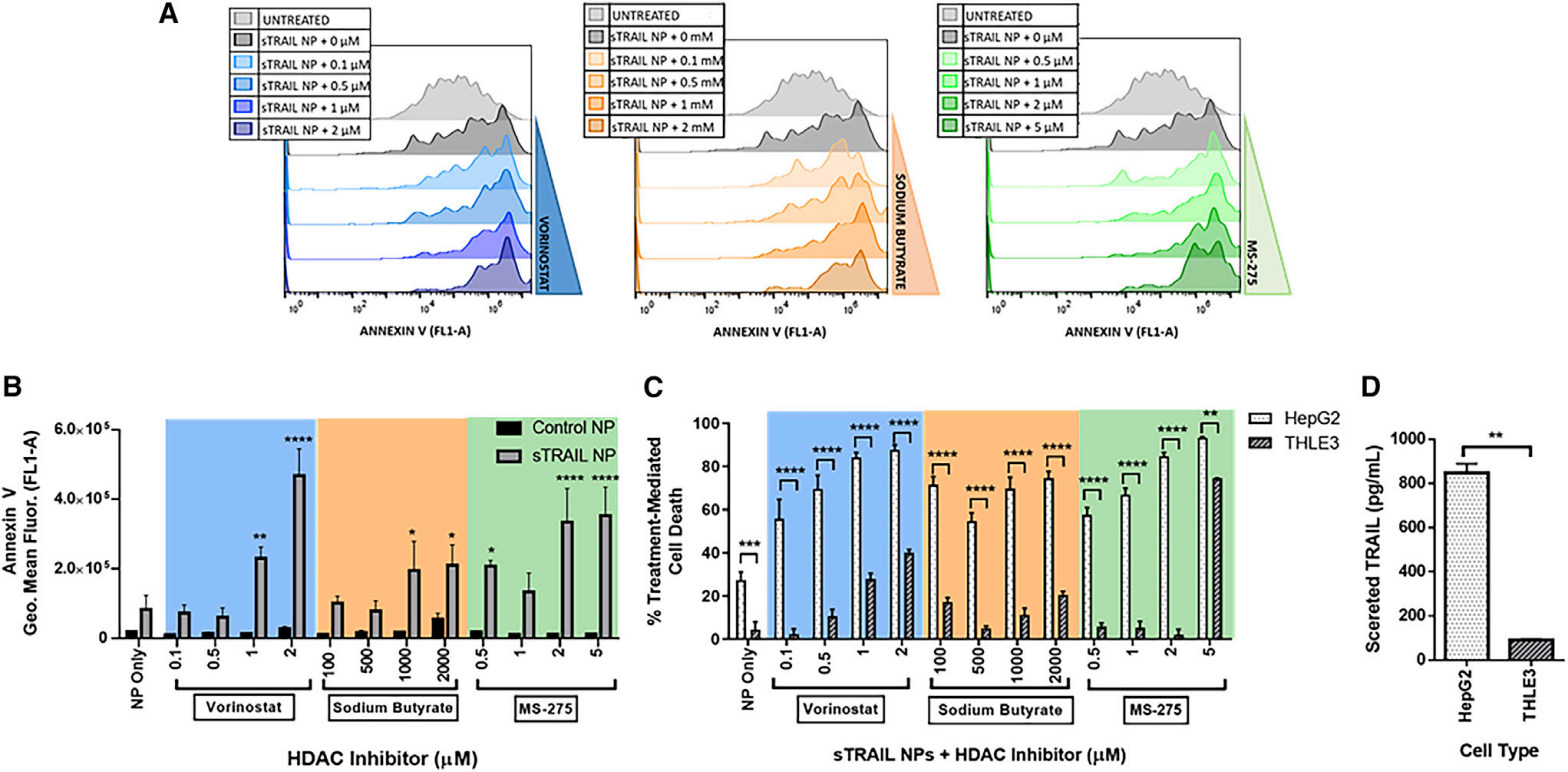
Combination treatment with sTRAIL NPs and HDAC inhibitors causes a dose-dependent and cancer-specific apoptosis (A) Histograms of Annexin V staining (FL1-A) in HepG2 cells transfected with sTRAIL NPs and treated with various doses of the HDAC inhibitors vorinostat, sodium butyrate, and MS-275. (B) Quantification of Annexin V staining results by flow cytometry, showing HepG2 cells treated with HDAC inhibitors and with PBAE 536 NPs containing GFP or sTRAIL. Comparisons between sTRAIL and control NP treatments were made by two-way ANOVA and Sidak’s multiple comparison test. (C) Cell death 48 h after transfection, normalized to negative controls treated with GFP NPs and corresponding HDAC inhibitor dose to calculate treatment-mediated cell death. Comparisons between HepG2 and THLE3 were made by two-way ANOVA and Sidak’s multiple comparison test. (D) Secreted human TRAIL protein in sTRAIL-transfected HepG2 HCC cells and THLE3 healthy hepatocytes, measured by ELISA 48 h after transfection. Comparison of TRAIL secretion between HepG2 and THLE3 was made by unpaired t test with Welch’s correction. Data represented as mean ± SEM of n = 3 replicate wells. *∗*p < 0.05, *∗∗*p < 0.01, ∗∗∗∗p < 0.0001.

We evaluated apoptotic cell death by quantifying phosphatidyl serine expression on the outer cell membrane using Annexin V staining. Apoptosis in HepG2 cells increases with sTRAIL transfection, as indicated by a shift in the Annexin V histogram curve (Figure 4A). Annexin V staining further increases with higher HDAC inhibitor doses, confirming that TRAIL-mediated apoptosis is dose dependent with HDAC inhibitor concentration. This increased Annexin V staining is not observed in GFP-transfected HepG2 cells treated with HDAC inhibitors (Figure 4B). This confirms that treatment-mediated apoptosis is due to a synergistic effect from secreted TRAIL and HDAC inhibitors, not from the inhibitors alone or NP cytotoxicity.

Next, we evaluated the cancer-specificity and off-target toxicity of our combination approach by comparing treatment effect in HepG2 HCC cells to THLE3 hepatocytes. While TRAIL-induced apoptosis is generally considered cancer-specific, there is evidence that modified versions of TRAIL protein may cause hepatotoxicity in healthy human cells.^41^ Further, certain sensitizing drugs, including HDAC inhibitors, have shown hepatotoxicity when combined with TRAIL therapy.^42^ We treated both HepG2 HCC and THLE3 hepatocyte cell lines with sTRAIL NPs and HDAC inhibitors, then compared the treatment-mediated cell death in the healthy and cancer cell types (Figure 4C). sTRAIL NPs alone caused very low toxicity in hepatocytes, with 5% cell death in THLE3 cells compared with 27% in HepG2 cells. When HDAC inhibitors were used to sensitize the cells, treatment-mediated cell death was increased to 80%–90%. The combination treatment was significantly more toxic to HepG2 cells over THLE3 at all HDAC inhibitor doses, with up to 40-fold higher cell death in the cancer cells. At higher HDAC inhibitor concentrations, cell death was increased in both HepG2 and THLE3 cells, indicating that the sensitizer dose must be carefully balanced to achieve a potent anticancer effect but not cause hepatotoxicity.

To deduce a mechanism of cancer-specificity, we used an ELISA to quantify TRAIL secretion from each cell type. HepG2 cells transfected with sTRAIL NPs secreted over 9 times more TRAIL protein than THLE3 cells (Figure 4D). To account for this difference in TRAIL secretion, THLE3 and HepG2 cells were treated with sTRAIL-conditioned media from transfected HepG2 cells. Without HDAC inhibitors, there was an 18% increase in HepG2 cell death with sTRAIL-conditioned media, indicating that the secreted TRAIL can potentiate a bystander effect to non-transfected cancer cells (Figure S7). This effect was dose-dependent with HDAC inhibitor concentration, with the greatest effect of 37% TRAIL-mediated cell death at 2 μM vorinostat. The cancer-specificity of the sTRAIL NP treatment is predominantly due to higher and preferential transfection of HCC cells by PBAE 536 NPs leading to cancer-specific apoptosis.

### Locally administered PBAE NPs enable DNA delivery to HepG2 xenograft tumors and slow tumor growth

**Figure 5:**
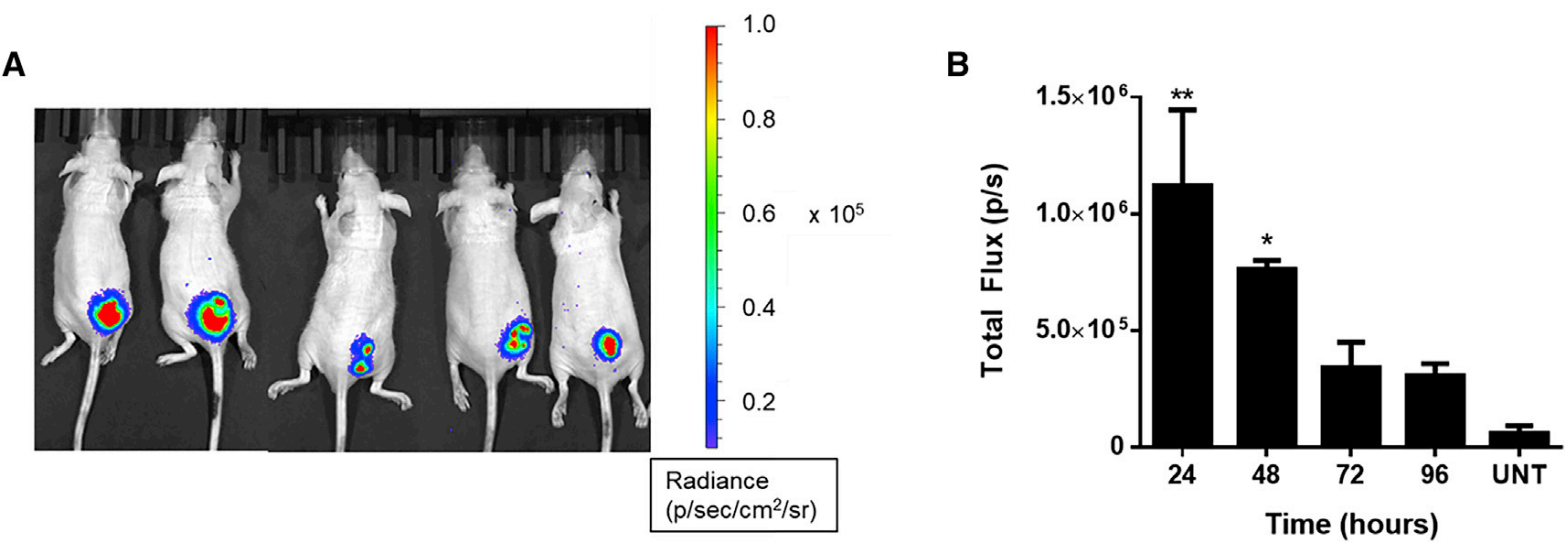
Intratumoral administration of PBAE 536 NPs results in strong gene expression in subcutaneous xenograft tumors (A) Bioluminescence images of subcutaneous HepG2 tumors 24 h after treatment with fLuc-PBAE 536 NPs. (B) Average bioluminescence over time in tumors injected with PBAE 536 NPs containing firefly luciferase plasmid DNA. Data represent mean ± SEM of 4–5 animals. Statistically significant differences between tumors treated and untreated tumors were calculated using one-way ANOVA with Dunnett’s post hoc test. *∗*p < 0.05, ∗∗p < 0.01.

To evaluate the translational potential of this approach *in vivo*, we assessed delivery of a reporter gene to HCC xenograft tumors. HepG2 tumors were established in the hind flank of athymic nude mice. PBAE NPs carrying a plasmid encoding firefly luciferase (fLuc)^43^ were injected directly into the tumors at a 5 μg DNA dose. 24, 48, 72, and 96 h later, D-luciferin was administered, and *in vivo* bioluminescence imaging was performed. Strong Luc expression was detected as early as 24 h after treatment (Figure 5A). The average total flux across the tumor area was significantly higher than background, with an average total flux of 1.1 ± 0.3 × 10^6^ p/s (Figure 5B). Radiance was greatest at 24 h and then decreased over the course of 4 days, with the average total flux still 5-fold higher than background 96 h after injection. All *in vivo* imaging can be found in Figure S8. This study confirmed that PBAE 536 NPs enable efficient gene delivery to HepG2 tumors *in vivo*.

**Figure 6:**
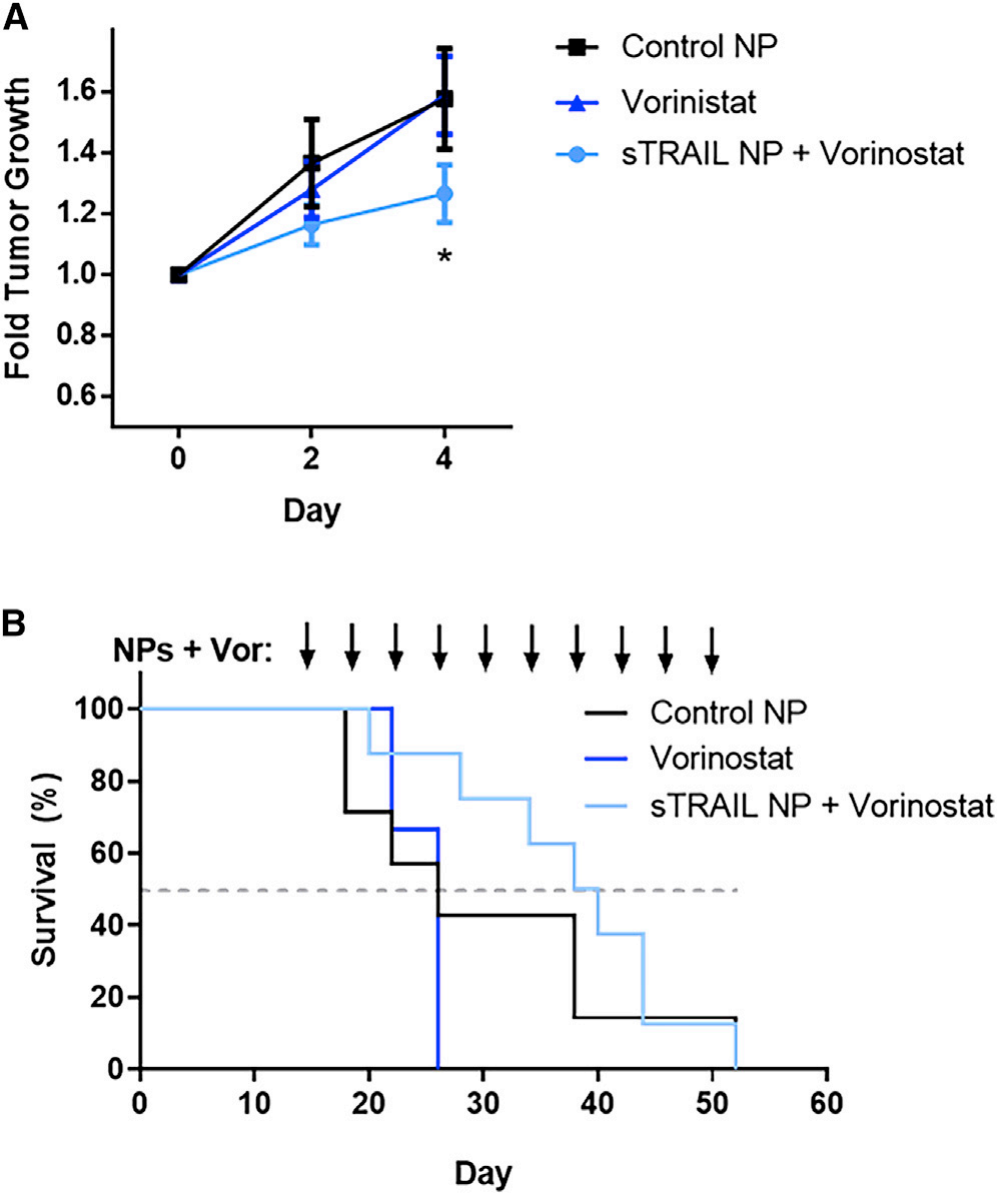
sTRAIL NPs administered intratumorally with systemic vorinostat slow the growth of HepG2 subcutaneous xenografts (A) Normalized subcutaneous HepG2 tumor size over 4 days in animals treated with control NPs only (N = 7), vorinostat only (N = 3), and sTRAIL NPs with vorinostat (N = 8). Data is represented as mean ± SEM. Significant differences in average tumor size between groups determined by two-way ANOVA with Sidak correction for multiple comparisons. *∗*p < 0.05 (B) Kaplan-Meyer survival curves of tumor-bearing mice. Dotted line drawn to indicate 50% survival.

Finally, to evaluate the *in vivo* efficacy of this system, we randomly assigned subcutaneous xenograft HepG2 tumors to one of three treatment arms: control fLuc NPs, IV vorinostat, or sTRAIL NPs with IV vorinostat. NPs harboring fLuc plasmid were selected to control for potential immunogenicity or toxicity from expression of a foreign protein and isolate the TRAIL-mediated anti-tumor effect.^44^ Vorinostat was selected for *in vivo* testing because it showed promising *in vitro* anti-cancer activity in combination with sTRAIL NPs, and this drug is already clinically approved for human use to treat cutaneous T cell lymphoma.^45^ Starting 14 days after tumor implantation, every 4 days animals received intratumoral injection of PBAE NPs at a 10 μg DNA dose and/or intravenous administration of vorinostat at an estimated blood concentration of 10 μM. Tumor measurements over the first 4 days of treatment indicated that sTRAIL NPs with vorinostat showed significantly slowed growth compared with animals receiving control NPs (Figure 6A). No antitumor effect was observed from vorinostat alone. Median survival with sTRAIL NPs and vorinostat treatment was 39 days, compared to a median of 26 days in the control NP alone and vorinostat alone groups (Figure 6B), which is an increase of 50%.

## Discussion

Here we describe a novel non-viral TRAIL gene therapy that induces potent and cancer-specific cell death in HCC. We utilize PBAE 536 NPs, a gene delivery vehicle that facilitates cancer-specific transfection in a wide range of HCC cell lines.^27^ Structurally similar polymers have been optimized to specifically transfect brain, lung, and breast cancers, showing the versatility of this strategy in heterogeneous tumors and diverse cancer types.^28^^,^^46^, ^47^, ^48^ While the mechanism of cancer-specific uptake and transfection is not fully understood, work by Zamboni et al.^27^ indicates that it is not driven by differences in cell division rate or NP uptake alone. Our group found that changes in PBAE endcap structure bias the route of endocytosis, thereby influencing NP uptake and transfection.^25^^,^^49^^,^^50^ Kim et al.^48^ showed that endocytosis route is predictive of transfection efficacy, with clathrin-mediated endocytosis of PBAE NPs disproportionately responsible for transfection over caveolae-mediated endocytosis and macropinocytosis. Because endocytosis is one of many pathways frequently dysregulated in cancer, a link between material properties and biological mechanism may provide a means for rational design of cancer-targeting biomaterials.^51^

A non-viral plasmid was constructed to enable exogenous expression of a secretable trimeric TRAIL protein. We show that the HCC-targeted PBAE 536 NPs enabled therapeutic delivery of the new plasmid encoding for sTRAIL. Transfection of HepG2 HCC cells with sTRAIL plasmid results in high levels of TRAIL protein secretion, enabling cell killing of both transfected cells and non-transfected bystander cancer cells. Because non-viral delivery vehicles tend to have lower transfection efficacy than viral methods, and penetration of tumors can be difficult, this bystander effect is critical to achieve potent tumor killing. In this case, PBAE NPs enable 53% transfection but >80% cell death by sTRAIL gene therapy. Corroborative results by Shah et al.^29^ demonstrated that a cDNA encoding sTRAIL protein delivered virally to glioma cells also induced apoptosis in non-infected bystander cells. Due to this bystander effect, transfection with sTRAIL cDNA produces a significantly enhanced therapeutic effect over non-secreted TRAIL. In contrast to previous work, the current research demonstrates that sTRAIL can be delivered efficaciously through non-viral NPs.

One major barrier to TRAIL therapy for cancer is the well-documented innate and acquired TRAIL resistance in certain tumors.^52^ We used HDAC inhibitors vorinostat, sodium butyrate, and MS-275 to sensitize HepG2 cells to TRAIL gene therapy. With increasing concentrations of these small molecule drugs, there is a synergistic and dose-dependent increase in TRAIL-mediated cell death and upregulation of phosphatidyl serine on the outer cell membrane. Studies have shown that HDAC inhibitors caused upregulation of death receptors and Bcl-2 family proapoptotic factors while simultaneously downregulating inhibitors of apoptosis.^53^ We found that vorinostat treatment induced upregulation of death receptor expression in HepG2 cells, suggesting a mechanism for the observed increase in TRAIL sensitivity. While it remains to be seen whether this sensitizing mechanism is conserved between cancer types, these results are further evidence that clinically approved HDAC inhibitors may improve clinical efficacy of TRAIL therapies, including gene therapy. Future studies of sTRAIL combination therapy in cells derived from primary human tumors would be valuable to better understand the heterogeneity of TRAIL resistance in a clinical setting. These studies may also reveal biomarkers that can be used to select for patients who are more likely to respond to TRAIL treatment in a personalized medicine approach.^46^

TRAIL is known to selectively initiate apoptosis in cancer cells while sparing normal cells, which has underpinned its investigation as a targeted cancer therapy. Interestingly, we find that TRAIL-conditioned media combined with HDAC inhibitors may have equivalent or greater toxicity to healthy hepatocytes than to HCC cells. This contrasts with historical studies showing that TRAIL has minimal off-target toxicity to normal cells.^6^^,^^54^ However, there have been published reports of elevated TRAIL toxicity in human hepatocytes relative to rodent or primate cells, which suggests that TRAIL sensitivity in normal cells is species-specific.^55^ Additionally, combination treatment with sensitizing drugs has been reported to also sensitize healthy cells to TRAIL.^56^ Therefore, the hepatotoxicity observed in these studies is consistent with the established literature. Death receptors have also been implicated in liver injury, including steatohepatitis and hepatitis.^57^ Elevated DR4 and DR5 expression in these conditions result in increased TRAIL-mediated hepatocyte apoptosis. Because liver tumors often develop in patients with underlying liver disease, this highlights the importance of employing cancer-targeted delivery vehicles for TRAIL therapy to minimize off-target hepatotoxicity.^58^ In further development of sTRAIL NPs in orthotopic tumor models, it will be essential to evaluate the bystander effect to healthy hepatocytes and closely monitor toxicity to the surrounding liver tissue.

The *in vivo* results demonstrate that PBAE NPs are effective for gene delivery to solid HCC tumors. A sTRAIL plasmid was constructed and validated to release sTRAIL to the supernatant, cause apoptosis of liver cancer cells, and synergize with small molecule drugs. PBAE NPs were validated to selectively transfect liver cancer cells over healthy hepatocytes, and via delivery of sTRAIL, enable liver cancer cell-specific killing. PBAE NPs shuttling a cDNA encoding for sTRAIL slow HepG2 tumor growth when combined with systemically administered vorinostat. To our knowledge, this is the first demonstrated application of PBAE NPs for the treatment of liver cancer. Successful development of a potent non-viral TRAIL gene therapy has broad implications for cancer treatment. While subcutaneous tumors were used for these studies to allow direct access for intratumoral injection and measurement, they fail to accurately recapitulate the tumor microenvironment and interactions between cancer and stromal cells.^59^ Further, intratumoral injection of therapeutic agents is not feasible in a clinical setting, and systemic delivery introduces additional delivery barriers, including serum aggregation or degradation, macrophage uptake, and intratumoral pressure.^60^ Therefore, future work should employ orthotopic HCC tumor models to study biodistribution, off-target transfection, and systemic toxicity of PBAE NPs.

Because death receptors are upregulated on many cancer types, sTRAIL gene therapy is not limited to HCC. Tzeng et al.^31^ showed that PBAE NPs encoding for membrane-expressed TRAIL selectively induced >60% cell death in lung and pancreatic cancers, suggesting that these cancer types may be suitable future targets for sTRAIL NPs. These NPs also have a promising safety profile, due to their rapid degradation in physiological conditions. Here we show that PBAE 536 NPs are non-toxic to hepatocytes, and PBAE NPs also have been proven safe in brain and retinal tissues *in vivo*.^61^^,^^62^ Further, PBAE 536 NPs are within the size range to potentially passively target tumors by the EPR effect. In addition, recent work shows that PEG-conjugated PBAE NPs have enhanced stability and tumor penetrating properties.^63^ Thus, non-viral PBAE sTRAIL NPs, with favorable pharmacokinetics and enabling sustained *in vivo* gene expression, may have therapeutic promise for various solid tumors.

### Conclusions

Non-viral delivery of cDNA encoding for sTRAIL in combination with HDAC inhibitors results in *in vitro* and *in vivo* anti-tumor efficacy in HCC with minimal toxicity to human hepatocytes. Considering the safety benefits of utilizing a non-viral gene therapy vector, this approach should be investigated further for clinical use.

## Materials and methods

### Polymer synthesis

1,5-pentanediol diacrylate (B5; Monomer-Polymer and Dajac Labs, Trevose, PA, USA), and 3-amino-1-propanol (S3; Alfa Aesar, Ward Hill, MA, USA) were combined in a 1:1.1 molar ratio of backbone to sidechain monomer and polymerized at 90°C under stirring for 24 h. The resulting acrylate-terminated polymer (B5S3) was dissolved in tetrahydrofuran, and 2-(3-aminopropylamino)ethanol (E6; Sigma-Aldrich, St. Louis, MO, USA) was added at a 10-fold molar excess. The end capping reaction was allowed to proceed for 1 h at room temperature (RT) under stirring. Endcapped PBAE polymer 536 was purified twice in diethyl ether to remove unreacted monomer and short oligomers and then dried under desiccant for approximately 48 h to remove traces of ether. PBAE 536 was dissolved in anhydrous DMSO and stored at −20°C with desiccant. Molecular weight was characterized by Gel Permeation Chromatography (GPC, Waters 2414 Refractive Index Detector, Milford, MA, USA).

### Plasmid DNAs

pEGFP-N1 (eGFP) DNA was purchased from Clontech Laboratories (Mountain View, CA, USA) and amplified by Aldevron (Fargo, ND, USA). pEGFP-TRAIL (mTRAIL) was a gift from Bingliang Fang (Addgene Plasmid #10953; Cambridge, MA, USA). Luciferase-pcDNA3 was a gift from William Kaelin (Addgene plasmid # 18964) and amplified by Aldevron (Fargo, ND, USA).

A non-viral plasmid encoding sTRAIL was designed and synthesized based on published work.^29^ Coding sequences from the extracellular domain of Flt3L (amino acids, aa 1–81), an isoleucine zipper sequence from the pFETZ vector, and the apoptosis-inducing sequence derived from the N terminus of the human TRAIL sequence (aa 114–281) were combined in-frame. sTRAIL cDNA was synthesized using custom gene synthesis from Integrated DNA Technologies (IDT; Coralville, IA). The empty pN3-Control backbone was a gift from Guntram Suske (Addgene plasmid # 24544). sTRAIL cDNAwas cloned into pN3-control backbone by restriction enzyme digest and amplified using ZymoPURE Plasmid Gigaprep kit (Zymo Research, Irvine, CA, USA).

### Nanoparticle synthesis and characterization

Plasmid DNA and PBAE 536 polymer were separately dissolved in pH 5 25 mM sodium acetate and combined at equal volumes, with a 1:25 mass ratio of polymer to DNA. NPs were allowed to assemble for 10 min and then diluted 5× or 10× in pH 7.4 PBS. Size was measured by DLS and zeta potential was measured by electrophoretic light scattering by a Malvern Zetasizer Nano ZS (Malvern Instruments, Malvern, UK). To measure encapsulation efficiency, we combined PBAE NPs with 6X loading dye without SDS. The samples were run through 0.8% agarose gel and using ethidium bromide staining and UV exposure to visualize the DNA bands.

### Cell culture

HepG2 and THLE3 cells were purchased from ATCC (Manassas, VA, USA) and cultured according to the vendor’s specifications. HepG2 cells were cultured in MEM media supplemented with 10% fetal bovine serum (FBS), 1% penicillin/streptomycin (Pen/Strep), 100 μM MEM non-essential amino acids, and 1 mM sodium pyruvate. THLE3 were cultured in bronchial epithelial cell growth medium (BEBM) supplemented with 10% FBS, 1% Pen/Strep, 5 ng/mL human epithelial growth factor (EGF), 70 ng/mL O-phosphorylethanolamine, and the BEGM bullet kit (Lonza/Clonetics Corporation, Walkersville, MD, USA) except Gentamycin-Amphotericin and Epinephrine. THLE3 cells were grown on plates and flasks coated with 0.01 mg/mL fibronectin, 0.03 mg/mL bovine collagen type I, and 0.01 mg/mL bovine serum albumin dissolved in culture media. Coating was performed overnight at 37°C.

### *In vitro* transfection

Cells were plated in tissue culture treated 96-well plates at 10,000 cells per well and allowed to attach overnight. PBAE 536 NPs were freshly prepared and added to wells at a final DNA dose of 0.6 μg per well. Cells were incubated with the NPs for 2 h at 37°C, then replenished with cell culture media. In HDAC inhibitor experiments, vorinostat (Adipogen, San Diego, CA, USA), sodium butyrate (Sigma-Aldrich, St. Louis, MO, USA), and MS-275 (Sigma-Aldrich, St. Louis, MO, USA) were diluted from stock solutions in cell culture media and added to cells after NP incubation. For media transfer studies in Figure S7, transfected cells were cultured for 48 h, then conditioned media was spun down at 300 rcf for 5 min to remove dead cells and debris. HDAC inhibitors were added to conditioned media and transferred to non-transfected HepG2 or THLE3 cells, seeded 24 h prior.

### Viability and transfection analysis

Brightfield images were acquired 48 h after transfection using a Zeiss (Oberkochen, Germany) Axio Observer fluorescence microscope at 10X magnification. Flow cytometry was performed 48 h after transfection using a BD Accuri C6 Flow Cytometer (BD Biosciences, San Jose, CA, USA) outfitted with a HyperCyt autosampler (IntelliCyt Corporation, Albuquerque, NM, USA) to enable high-throughput analysis. Cells were prepared for flow by detaching in 0.25% trypsin-EDTA (HepG2) or 0.05% trypsin-EDTA (THLE3), then resuspending in 30 μL of 2% FBS solution in 1X PBS. To assess viability, we also stained cells with a 1:200 dilution of propidium iodide (PI). Data was analyzed using FlowJo v10 (Ashland, OR, USA). Events were gated on FSC-H and SSC-H to identify the cell population, then on FSC-H and FSC-A to exclude doublets. For transfection toxicity analysis, the percentage of cells was calculated by determining the percentage of cells stained positive for PI. For GFP transfection analysis, dead cells that stained PI+ (FL3-A) cells were also excluded. Percentage of GFP-positive cells and normalized geometric mean fluorescence (FL1-A) were calculated. For TRAIL efficacy studies, viability was measured 24 h after transfection using an MTT cell proliferation assay (Promega, Madison, WI, USA).

### Western blot

Total protein was extracted from tumor cells 24 h following treatment with DMSO (Sigma Aldrich, USA), 0.1 μM and 0.5 μM of Vorinostat (Thermo Fisher Scientific, USA), and quantified using Bradford protein assay (Bio-Rad, USA). 10 μg of the protein lysates from these samples were loaded onto 10% Mini-PROTEAN TGX Precast Protein Gels (Bio-Rad, USA) and conducted at 110 V for 90 min. Following the separation step, proteins were transferred to a polyvinylidene fluoride (PVDF) membrane at 100 V for 1 h. Membranes were blocked with 5% dry milk or 5% bovine serum albumin (BSA) and 0.1% Tween-20 in TBS for 1 h at RT, and incubated with primary antibodies in TBS-T overnight at 4°C. After treatment with HRP-conjugated secondary antibodies in TBS-T for 1 h at RT, membranes were developed with Super Signal West Pico system (Thermo Fisher Scientific, USA) and then signals were visualized using autoradiographic films. Antibodies used: DR4 (ProSci 1139), DR5 (ProSci 2019), Vinculin (Sigma V4505), anti-rabbit immunoglobulin G (IgG) HRP-linked (Cell Signaling Technologies 7074), and goat anti-mouse IgG H&L (HRP; Abcam ab97023). Vinculin was used as a loading and internal control.

### Flow cytometry analysis of cell surface receptors

Cells were harvested with trypsin, washed, and resuspended in PBS, and then stained with Zombie UV Fixable Viability Kit (BioLegend 423107) for 30 min at RT, followed by a wash and then incubated with primary antibody for 60 min at 4°C in the dark; then samples were washed and incubated with secondary conjugated antibody for 60 min at 4°C in the dark. Samples were then resuspended in PBS with 1% FBS (Gibco, USA). Flow cytometry was performed using FACS Fortessa (BD) cell sorter and data was analyzed using FlowJo (BD). All washes were performed with PBS. Antibodies used were as follows: APC anti-human CD262 (DR5, TRAIL-R2; BioLegend 307407), DR4 (Santa Cruz Biotechnology, sc-32255), and IgG1 cross-adsorbed goat anti-mouse, Alexa Fluor 488 (Invitrogen A21121).

### Annexin V stain

Annexin V, fluorescein isothiocyanate (FITC) conjugate was purchased from BioLegend (San Diego, CA, USA). 48 h after transfection and/or sensitizer treatment, cells were resuspended in 100 μL staining buffer with 1:200 dilution of PI. 5 μL of Annexin V stain was added per well, and cells were incubated at RT for 15 min. Cells were spun down, washed once in 1X PBS, then resuspended in 2% FBS for flow cytometry, as described above. Geometric mean fluorescence (FL1-A) was calculated and reported. Histograms from representative wells were created using FlowJo v10 (Ashland, OR, USA).

### TRAIL ELISA

To collect lysates, we washed cells 3X with ice-cold PBS and then treated them with cell extraction buffer (Invitrogen, Carlsbad, CA, USA) supplemented with 5% protease inhibitor (Sigma Aldrich, St. Louis, MO, USA) for 30 min in ice. Lysates were thoroughly mixed by pipetting and then centrifuged for 10 min at 13,000 rpm at 4°C. Supernatants were transferred and stored at −80°C until used. Conditioned cell culture media was collected and centrifuged for 10 min at 1,500 rpm at 4°C. Supernatants were also stored at −80°C. Human TRAIL ELISA was purchased from Invitrogen (Carlsbad, CA, USA), and the assay was run according to manufacturer’s instructions. Human TRAIL protein dilutions were used as standards and run in duplicate. Absorbance was measured at 450 nm using a Biotek Synergy 2 plate reader (Winooski, VT, USA). Results from the standards were plotted and fit to a 5-parameter fit curve in GraphPad Prism 6 (San Diego, CA, USA). Sample concentrations were calculated by interpolating the fit curve.

Total protein contents of cell lysates were measured using a Pierce BCA Protein Assay Kit (Waltham, MA, USA). BSA standards were prepared and run in duplicate to create a standard curve. The kit was used following manufacturer’s instructions, and absorbance was measured at 562 nm on a Biotek Synergy 2 plate reader (Winooski, VT, USA). Absorbance measurements from the BSA standards were plotted and fit to a 5-parameter fit curve in Graphpad Prism 6 (San Diego, CA, USA). Protein concentrations of samples were determined by interpolating the fit curve. TRAIL concentration in each sample was normalized to total protein content by BCA and was reported as pg of TRAIL per μg total protein.

### Animal models

All *in vivo* procedures were approved and overseen by the Johns Hopkins Institutional Animal Care and Use Committee (IACUC). To establish xenograft tumors, we resuspended 1 million HepG2 cells in 100 μL of 50% Matrigel matrix HC (Corning, Corning, NY, USA) and 50% HBSS. Cells were injected subcutaneously in the hind flank of female 6- to 8-week-old athymic nude mice (The Jackson Laboratory, Bar Harbor, ME, USA). During implantation, animals were anesthetized with 2.5% isoflurane in oxygen. Tumors developed in ∼80% of mice after 14 days.

### *In vivo* gene delivery to SC tumors

To make NPs for *in vivo* gene delivery, we diluted PBAE 536 in pH 7.4 sodium acetate and then added fLuc plasmid for a final DNA concentration of 0.1 μg/μL. The polymer to DNA weight ratio was maintained at 25 w/w, and final sodium acetate concentration was 25 mM. NPs were stored at −80°C and thawed immediately prior to injection. Animals were anesthetized under isoflurane, and 50 μL of NPs were injected into the tumor using an insulin syringe, for a final 10 μg DNA dose. After 24, 48, 72, and 96 h, live *in vivo* imaging was performed using an IVIS Spectrum imaging system (Perkin Elmer, Waltham, MA, USA). 150 mg/kg D-luciferin (Gold Biotechnology, St. Louis, MO, USA) was administered intraperitoneally to mice and then imaging was performed 10 min later. Images were analyzed across regions of interest (ROI) using Living Image software (Perkin Elmer, Waltham, MA, USA).

### Anti-tumor efficacy and survival study

Mice were implanted with HepG2 tumors as previously described. PBAE NPs were synthesized with fLuc or sTRAIL plasmid in pH 7.4 sodium acetate at a DNA dose of 0.2 μg/μL and stored at −80°C. 14 days after tumor implantation, mice were randomized to three groups: (1) fLuc (control) NP + vehicle (n = 7), (2) vorinostat only (n = 3), and (3) sTRAIL NP + vorinostat (n = 8). Every 4 days, beginning on day 14, mice received intratumoral injections of NPs and retroorbital injections of 100 μL vehicle or 150 μM vorinostat. Tumor dimensions were measured every other day using calipers, and area was calculated by multiplying the longest dimension (length) by its perpendicular width. An animal was sacrificed when its tumor area grew larger than 200 mm^2^.

### Statistical analysis

All data are presented as a mean ± standard error of replicate tests. Comparisons between two groups were performed using a Student’s t test. Comparisons between multiple (>2) groups were performed using one-way ANOVA and Tukey or Dunnett post hoc test for multiple comparisons. Tests between groups with multiple factors were performed using two-way ANOVA and Sidak’s multiple comparison test. All statistical analyses were performed using GraphPad Prism 6 (San Diego, CA, USA).

## Supplemental information

Document S1. Figures S1–S8

Document S2. Article plus supplemental information
