# Supplementary material for: Poly(beta-amino ester) nanoparticles enable tumor-specific TRAIL secretion and a bystander effect to treat liver cancer
Source: Mol Ther Oncolytics. 2021 Apr 16;21:377–88. doi: 10.1016/j.omto.2021.04.004 (PMC8208964; doi:10.1016/j.omto.2021.04.004)
Supplement: Document S1. Figures S1–S8 [file mmc1.pdf]

OMTO, Volume *21*

Supplemental information

Poly(beta-amino ester) nanoparticles enable tumor-specific TRAIL secretion and a bystander effect to treat liver cancer

Hannah J. Vaughan, Camila G. Zamboni, Nicholas P. Radant, Pranshu Bhardwaj, Esther Revai Lechtich, Laboni F. Hassan, Khalid Shah, and Jordan J. Green

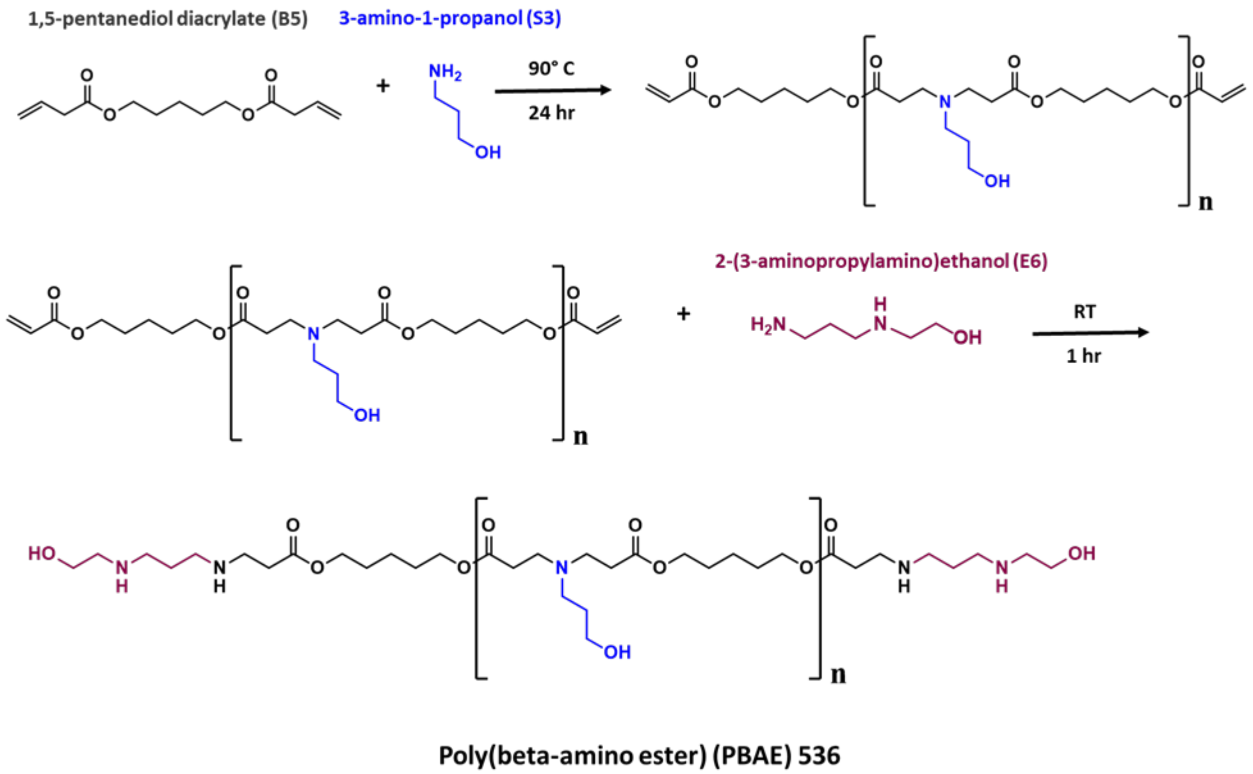

Supplemental Figure 1 Synthesis of polymer 2-((3-aminopropyl)amino)ethanol end-modified poly(1,5-pentanediol diacrylate-co-3-amino-1-propanol) (PBAE 536). 1,5-pentanediol diacrylate (B5) is combined with 3-amino-1-propanol (S3) at a 1:1.1 ratio of B5 to S3 and reacted neat under stirring at 90° C for 24 hours. The resulting acrylate-terminated polymer is dissolved in THF and reacted with 2-(3-aminopropylamino)ethanol (E6) in 10-fold excess for 1 hour at room temperature. Endcapped polymer is ether purified and dried under vacuum. Resulting polymer had a weight average of 5638 Da and polydispersity of 1.29 by GPC.

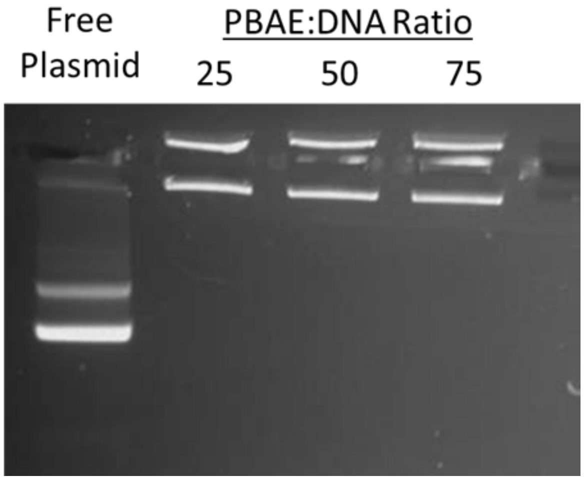

Supplemental Figure 2 Gel electrophoresis of 0.03 μg/μL eGFP plasmid and PBAE NPs formulated with eGFP plasmid and a 25, 50, and 75 w/w ratio of PBAE 536 polymer.

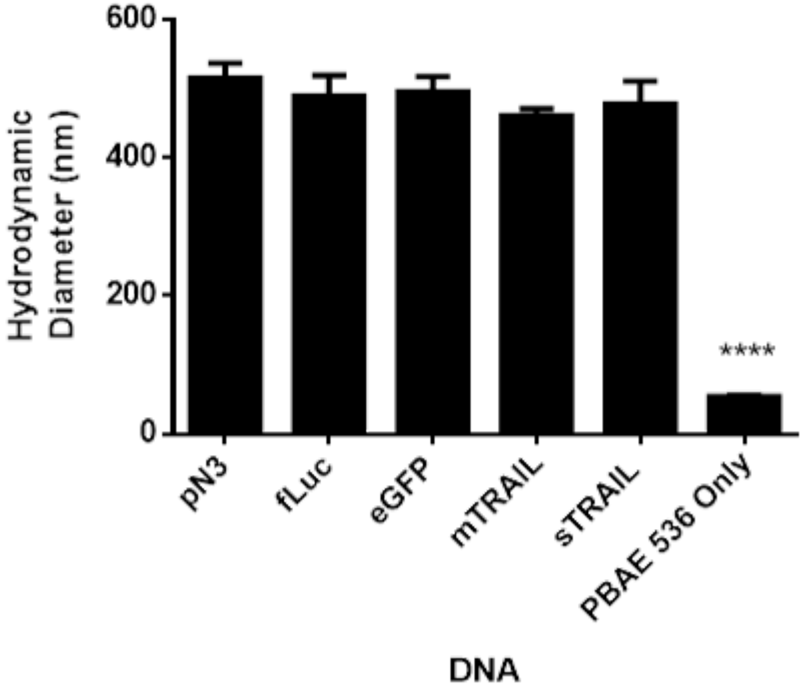

Supplemental Figure 3 Diameter of electrostatically complexed NPs comprised of PBAE 536 and plasmid DNA at a 25 weight ratio (w/w). Nanoparticles were synthesized in NaAc pH 7.4 and diluted in PBS pH 7.4 for DLS analysis.

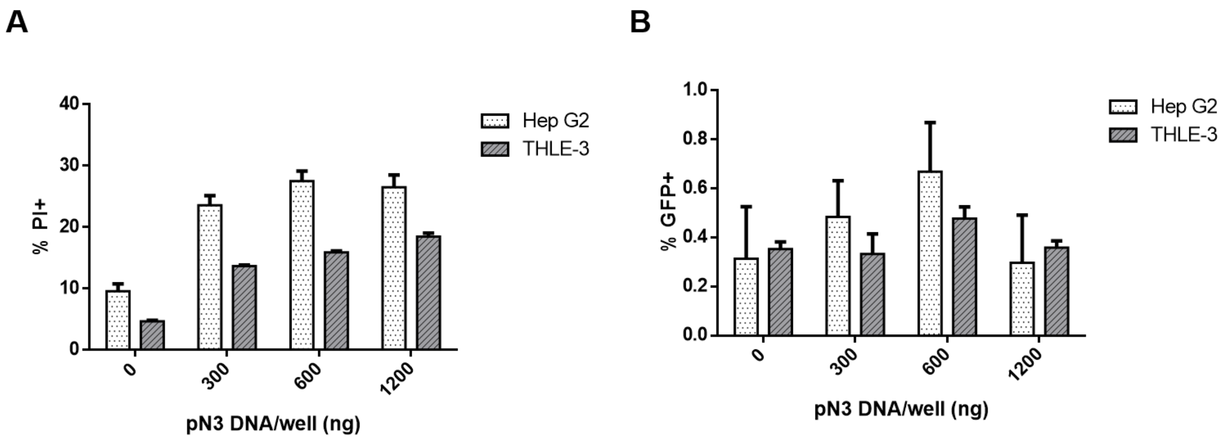

Supplemental Figure 4 A. Viability of HepG2 and THLE3 cells after treatment with PBAE 536 NPs at a range of pN3 DNA doses. Toxicity was determined by staining samples 1:200 with propidium iodide (PI) and measuring the percentage of PI+ cells by flow cytometry. **B.** *In vitro* pN3 transfection of HepG2 HCC cells and THLE3 hepatocytes transfected by PBAE 536 / pN3 NPs as measured by flow cytometry for GFP+.

sTRAIL Sequence:

ATGACAGTGCTGGCGCCAGCCTGGAGCCCAACA**ACCTATCTCCTCCTGCTGCT
GCTGAGCTCGGGACTCAGTGGGACCCAGGACTGCTCCTTCCAACACAGCCCCATC
TCCTCCGACTTCGCTGTCAAAATCCGTGAGCTGTCTGACTACCTGCTTCAAGATTAC
CCAGTCACCGTGGCCTCCAACCTGCAGGACGAGGAGCTCTGCGGGGGCCTCTGG
CGGCTGGTCCTGGCACAGCGCTGGATGGAGCGGCTCAAGACTGTCGCTGGGTCCA
AGATGCAAGGCTTGCTGGAGCGCGTGAACACGGAGATACACTTTGTCACCAAATGT
GCCTTTCAGCCCCCCCCTAGCTGTCTTCGCTTCGTCCAGACCAACATCTCCCGCCT
CCTGCAGGAGACCTCCGAGCAGCTGGTGGCGCTGAAGCCCTGGATCACTCGCCA
GAACTTCTCCCGGTGCCTGGAGCTGCAGTGTCAGCCCGACTCCTCAACCCTGCCA
CCCCCATGGAGTCCCCGGCCCCTGGAGGCCACAGCCCCGACAGCCCCGATGAAG
CAGATCGAGGACAAAATTGAGGAAATCCTGTCCAAGATTTACCACATCGAGAACGA
GATCGCCCGGATTAAGAAACTCATTGGCGAGAGGGAA**TTCACCTCTGAGGAAACCA
TTTCTACAGTTCAAGAAAAGCAACAAAATATTTCTCCCCTA**GTGAGAGAAAGAGGTCC
TCAGAGAGTAGCAGCTCACATAACTGGGACCAGAGGAAGAAGCAACACATTGTCC
TCTCCAAACTCCAAGAATGAAAAGGCTCTGGGCCGCAAAATAAACTCCTGGGAATC
ATCAAGGAGTGGGCATTCATTCCTGAGCAACTTGCACTTGAGGAATGGTGAACTGG
TCATCCATGAAAAAGGGTTTTACTACATCTATTCCCAAACATACTTTCGATTTCAGGA
GGAAATAAAAGAAAACACAAAGAACGACAAACAAATGGTCCAATATATTTACAAATA
CACAAGTTATCCTGACCCTATATTGTTGATGAAAAGTGCTAGAAATAGTTGTTGGTCT
AAAGATGCAGAATATGGACTCTATTCCATCTATCAAGGGGGAATATTTGAGCTTAAG
GAAAATGACAGAATTTTTGTTTCTGTAACAAATGAGCACTT**GATAGACATGGACCATG
AAGCCAGTTTTTTCGGGGCCTTTTTAGTTGGCTAA

MTVLAPAWSPT**TYLLLLLLLSSGLSGTQDCSFQHSPISSDFAVKIRELSDYLLQDYPVTV
ASNLQDEELCGGLWRLVLAQRWMERLKTVAGSKMQGLLERVNTEIHFVTKCAFQPPP
SCLRFVQTNISRLLQETSEQLVALKPWITRQNFSRCLELQCQPDSSTLPPPWSPRPLEA
TAPTAPMKQIEDKIEEILSKIYHIENEIARIKKLIGERE**FTSEETISTVQEKQQNISPL**VRER
GPQRVAAHITGTRGRSNTLSSPNSKNEKALGRKINSWESSRSGHSFLSNLHLRNGELVI
HEKGFYYIYSQTYFRFQEEIKENTKNDKQMVQYIYKYTSYPDPILLMKSARNSCWSKDA
EYGLYSIYQGGIFELKENDRIFVSVTNEH**LIDMDHEASFFGAFLVG*

Flt3L (a.a. 1– 81)

Isoleucine Zipper

Human TRAIL N Terminus (a.a. 114 –281)

Supplemental Figure 5: **A.** DNA sequence for sTRAIL gene **B.** Protein translation of sTRAIL DNA

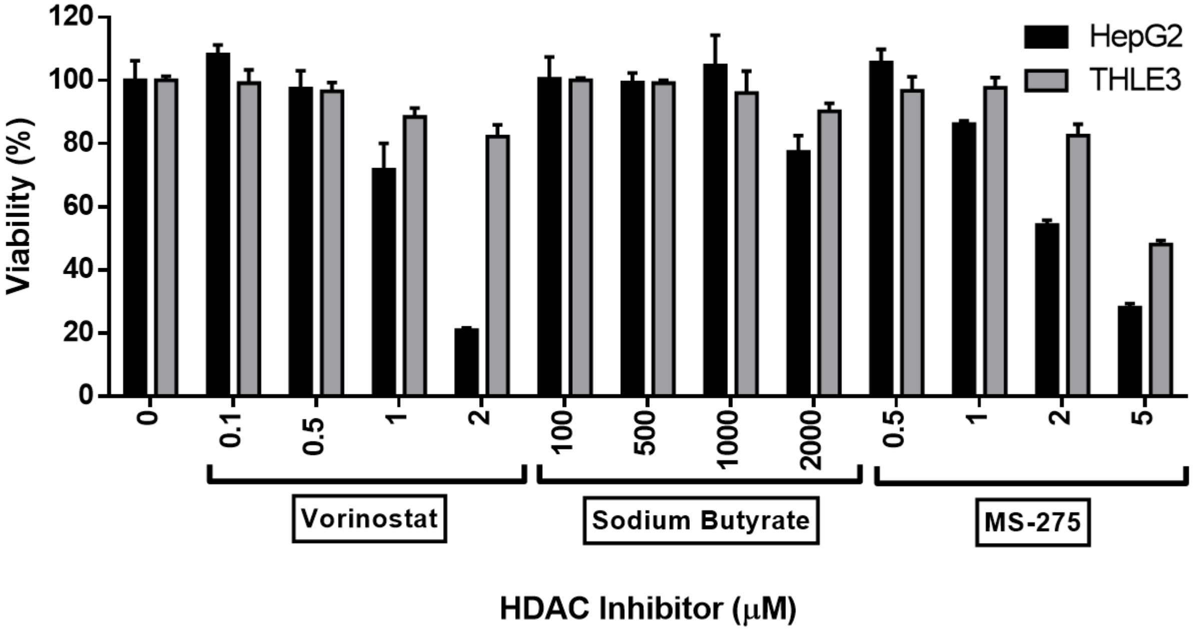

Supplemental Figure 6: Viability of HepG2 and THLE3 cells treated with HDAC inhibitors for 48 hours, measured by MTT.

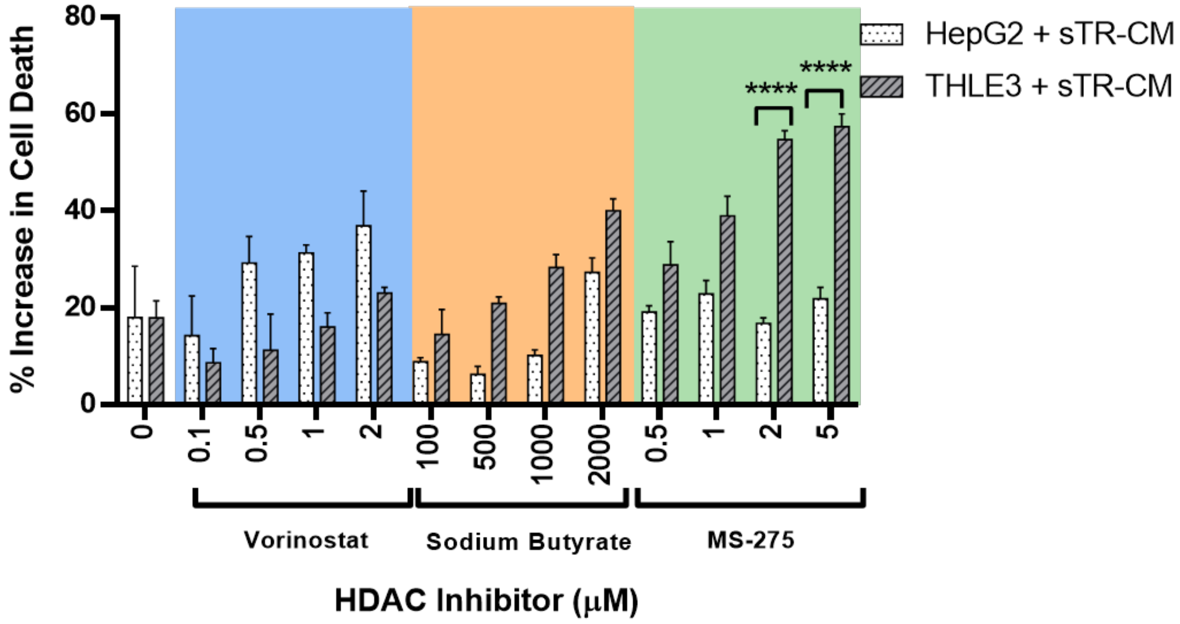

Supplemental Figure 7. Effects of sTRAIL Conditioned Media. Non-transfected HepG2 and THLE3 cells were treated with conditioned media from sTRAIL-transfected HepG2 cells (sTR-CM). After 48 hours, a viability assay was performed, and data was normalized to control wells with matched HDAC inhibitor exposure. All data is represented as mean ± SEM of n = 3 replicate wells. ***P < 0.01, ****P < 0.0001*.

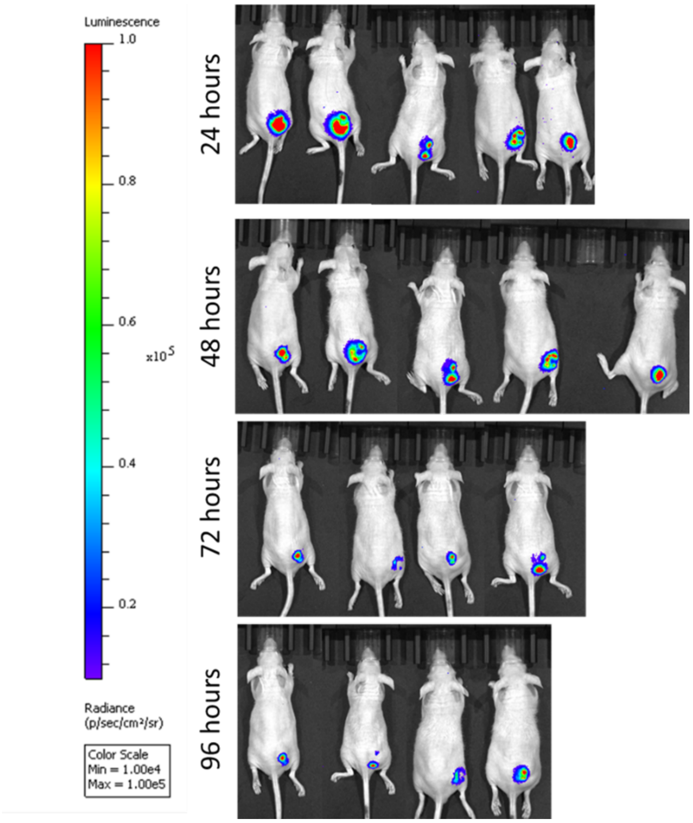

Supplemental Figure 8: Bioluminescence images of subcutaneous xenograft HepG2 tumors treated with intratumoral injections of PBAE 536 NPs containing firefly luciferase plasmid DNA. Images were captured 24, 48, 72, and 96 hours after NP treatment.
